# Data related to the PC_71_BM loading and it's impact on nanostructuring for blend of PBDTTT-EFT:PC_71_BM bulk heterojunction solar cell

**DOI:** 10.1016/j.dib.2017.11.076

**Published:** 2017-11-23

**Authors:** Soheil Komilian, Ochai Oklobia, Torfeh Sadat-Shafai

**Affiliations:** Staffordshire University, UK

## Abstract

The data included in this article is based on additional supporting information presented in our recent publication Komilian et al. [1]. The role of acceptor material (PC_71_BM) in restructuring copolymer PBDTTT-EFT from its relaxed pristine structure to interfaces suitable for exciton dissociation is discussed. The analysis of data indicates that the impact of acceptor material on nanostructuring initiates concurrent processes some of which supports and some impedes charge extractions. Therefore, this manuscript is designed to identify these processes and give and account of their impact on power conversion efficiency.

**Specifications Table**

**Table:** 

Subject area	Physics, Chemistry, Electrical/Electronic
More specific subject area	Photovoltaic/Polymeric solar cells
Type of data	Table, image and figure
How data was acquired	For IV measurements, Keithley 2400 source meter is used.
	Raman spectroscopy data were collected using Renishaw inVia. The device morphology was performed using Agilent AFM 5400 series.
Data format	Raw/analyzed data.
Experimental factors	Prior to fabrication of the active layer the substrate were transferred to a nitrogen glove box maintained at 0.1 ppm for O2 and H2O level left for 30 min. PEDOT: PSS was filtered and then spin coated onto the substrate at a speed of 5,000 rpm for 30 s and then baked on a hotplate at 120 °C for 10 min. PBDTTT-EFT: PC71BM with concentration of 25 mg mL^−1^ in 1,2-dichlorobenzene but with blend ratios of 1:0.5, 1:1, 1:1.15, 1:2 and 1:3 were produced. After fabrication of the active layer, substrates then vacuum dried for 5 min at −100 kPa ready for surface washing with 60 µL of Ethanol and drop cast at 4000 rpm for 30 s.
Experimental features	The active area for each device was 0.13 cm^2^, illuminated through a shadow mask under 1 SUN condition using 1.5 AMG filter (LOT-LSZ389) and xenon arc lamp solar simulator (LOT-LS0306). *I*–*V* characteristics were collected using Keithley 2400 source meter. To test the accuracy of solar simulator, silicon reference solar cell (LOT-LS0041) which its accuracy is certified by National renewable energy laboratory was used to adjust the input power. Raman and PL measurements were collected using (Renishaw inVia) with 685 excitation lasers. The device morphology was performed using Agilent AFM 5400 series.
Data source location	Thin films Laboratory, Staffordshire University. Stoke-On-Trent, UK.
Data accessibility	Information related to this article is available in appropriate tables.
Related research article	“Controlling intercalations of PBDTTT-EFT side chain to initiate suitable network for charge extraction in PBDTTT-EFT:PC_71_BM blended bulk heterojunction solar cell”

**Value of the Data**

• IV data presented in this article is based on several samples tested to identify the reproducibility of the results and providing further evidence to the conclusions made.
• Raman spectroscopy presented here identifies the impact of 685 nm laser used on collected data. The 785-nm laser used in the original manuscript can only provide evidence for PBDTTT-EFT vibrational mode impacted by PC_71_BM content.
• The analysis of surface morphology using AFM further supports our interpretation of donor acceptor Nano structuring based on device surface roughness.

## 1 Data

**Fig. 1:**
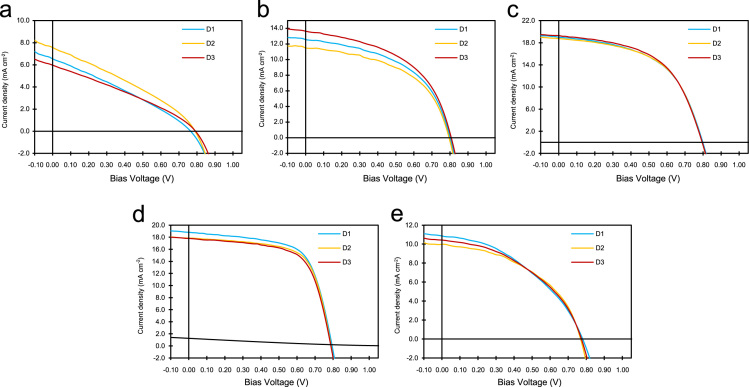
*J*–*V* characteristics of PBDTTT-EFT: PC_71_BM blend ratios representing device 1, 2 and 3 for each blend. **(a)** 1:0.5 blend ratio, **(b)** 1:1 blend ratio, **(c)** 1:1.5 blend ratio, **(d)** 1:2 blend ratio, **(e)** 1:3 blend ratio.

**Fig. 2:**
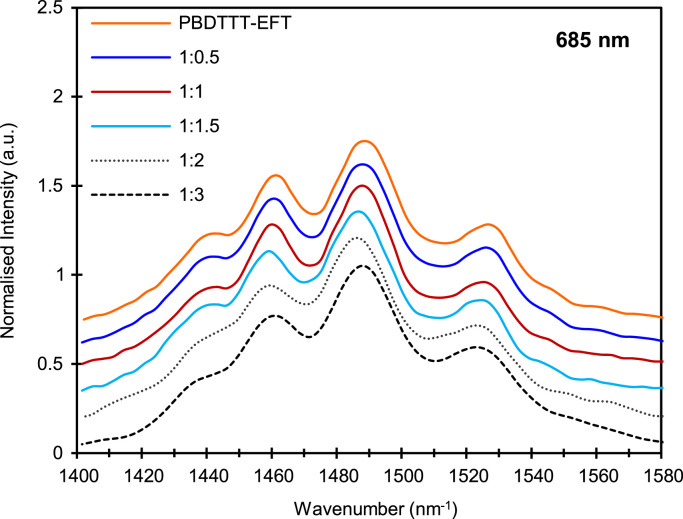
Raman Spectrum of PBDTTT-EFT: PC_71_BM at blend ratios collected using 685 nm excitation laser.

**Fig. 3:**
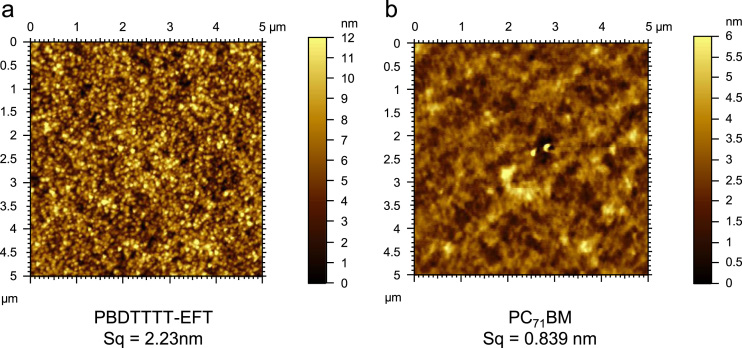
AFM Topography images of pristine PBDTTT-EFT **(a)** and PC_71_BM **(b)** respectively. PBDTTT-EFT surface roughness is Sq (RMS)=2.23 nm and PC_71_BM surface roughness is Sq (RMS)=0.839 nm.

In order to improve power conversion efficiency of blend of PBDTTT-EFT:PC_71_BM solar cell, it is better to have insight into how donor acceptor material interface will form within the thin film structure. To this end, purposely various percentage loading of PC_71_BM was introduced in the blend, to observe its impact on parameters such as power conversion efficiency, fill factor, Short circuit current density, open circuit voltage, device shunt and series resistance. The data presented here are based on additional experimental observation reported in our recent paper [1]. Electrical characterisation of these devices are shown in Fig. 1 and after analysis, summarized and presented in Table 1. To elaborate on the mechanisms for exciton creation, dissociations and finally charge extractions it is necessary to understand the role of PC_71_BM within the blend and how it may impact the polymer phase. To assist us with this, it was found necessary to have experimental evidences associated with these impacts. Also, experimental results associated with Raman and AFM are also included here to support readers to have clear picture to the approaches we have taken (Fig. 2, Fig. 3).

**Table 1:** Electrical parameters extracted from *J*–*V* curves in Fig. 1.

**Ratio**	**Device**	***J***_**sc**_**(mA cm**^**−2**^**)**	***V***_**oc**_**(*****V*****)**	**FF**	**PCE (%)**	***R***_**sh**_**(Ω cm**^**−2**^**)**	***R***_**s**_**(Ω cm**^**–2**^**)**
1:0.5	1	6.55	0.76	0.30	1.48	148.29	47.65
	2	7.56	0.79	0.32	1.88	145.56	39.6
	3	5.97	0.79	0.31	1.45	176.56	55.76
							
1:1	1	12.59	0.8	0.50	5.04	293.81	13.38
	2	11.55	0.79	0.51	4.65	336.65	13.15
	3	13.64	0.81	0.49	5.44	260.7	13.62
							
1:1.5	1	19.02	0.8	0.53	8.02	289.55	9.88
	2	18.75	0.79	0.54	8.05	315.21	9.55
	3	19.26	0.79	0.53	8.17	298.17	9.81
							
1:2	1	18.8	0.79	0.65	9.67	365.13	6.74
	2	17.85	0.79	0.67	9.37	569.23	6.18
	3	17.78	0.79	0.65	9.1	462.52	6.61
							
1:3	1	10.85	0.78	0.41	3.47	336.56	21.52
	2	9.97	0.77	0.46	3.53	402.07	13.41
	3	10.41	0.77	0.43	3.49	366.52	16.52

## 2 Experimental design, materials, and methods

Pre-fabricated ITO/glass substrates were first cleaned using ultra sonic bath with three step process; deionised water, acetone, isopropanol and left to dry in nitrogen environment [2]. PEDOT: PSS purchased from Ossila was filtered and then spin coated onto the substrate at a speed of 5,000 rpm for 30 s and then baked on a hotplate at 120 °C for 10 min. A DektakXT thickness profiler accurately measured the film to be 30–40 nm. The substrate was then transferred to a nitrogen glove box maintained at 0.1 ppm for O2 and H2O level left for 30 min before fabricating the active layer. PBDTTT-EFT: PC_71_BM (1-Material, used as received) with concentration of 25 mg mL^−1^ in 1,2-dichlorobenzene but with blend ratios of 1:0.5, 1:1, 1:1.15, 1:2 and 1:3 were produced. The pre-tested PBDTTT-EFT: PC_71_BM spin-casted at 600 rpm for 18 s resulted in a film thickness of ~ 100 nm. After fabrication of the active layer, substrates then vacuum dried for 5 min at −100 kPa ready for surface washing with 60 µL of Ethanol purchased from Sigma Aldrich and drop cast at 4000 rpm for 30 s. For fabrication of top electrodes, substrate was transferred to a metallisation rig (Auto 500) via an interconnecting chamber. First 10 nm of Calcium (sigma Aldrich) was fabricated at a rate of 0.1 nm s^−^^1^ using mask aligner. After a delay time of 5 minutes 100 nm of Aluminium was then deposited through the mask aligner at a rate of 0.1 nm s^−1^ for the first 20 nm and then at a rate of 0.5 nm s^−^^1^ for the remaining 80 nm. A microbalance quartz crystal monitor (Intellmetrics IL 150) was used to measure the deposition rate as well as the Aluminium thickness. During the deposition, the chamber vacuum was maintained below 10^−6^ Torr. For Raman and AFM studies, no metallisation were used.

The active area for each device was 0.13 cm^2^, illuminated through a shadow mask under 1 SUN condition using 1.5 AMG filter (LOT-LSZ389) and xenon arc lamp solar simulator (LOT-LS0306). *I*–*V* characteristics were collected using Keithley 2400 source meter. To test the accuracy of solar simulator, silicon reference solar cell (LOT-LS0041) which its accuracy is certified by National renewable energy laboratory was used to adjust the input power. The full experimental detail and the method of analysis and device characteristic parameters are presented in our previous communication [3].

Raman measurements were collected using (Renishaw inVia) with 685 nm excitation laser. The experimental set up and methodology is previously presented [4]. The device morphology was performed using Agilent AFM 5400 series. The analysis of data is based on film surface roughness previously reported [5].
