# Supplementary material for: Data related to the PC71BM loading and it's impact on nanostructuring for blend of PBDTTT-EFT:PC71BM bulk heterojunction solar cell
Source: Data Brief. 2017 Nov 23;16:506–10. doi: 10.1016/j.dib.2017.11.076 (PMC5734693; doi:10.1016/j.dib.2017.11.076)
Supplement: Supplementary file 1 — Supplementary material [file mmc1.docx]

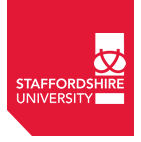


Professor T. Sadat-Shafai

School of Engineering

Thin Films Laboratory

Leak Road

Stoke-On-Trent

Staffordshire

ST4 2DE

[t.sadat-shafai@staffs.ac.uk](mailto:t.sadat-shafai@staffs.ac.uk)

+441785353475

**The impact of PC_71_BM loading on nanostructuring for blend of PBDTTT-EFT:PC_71_BM bulk heterojunction solar cell**

To whom it may concern

On behalf of the co-authors, I would like to state that there are no conflict of interest concerning the above manuscript.

Kind regards


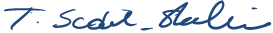


Professor T. Sadat-Shafai
